# Polyfunctional Nitriles in Organic Syntheses: A Novel Route to Aminopyrroles, Pyridazines and Pyrazolo[3,4-c]pyridazines

**DOI:** 10.3390/molecules14020798

**Published:** 2009-02-16

**Authors:** Saleh M. Al-Mousawi, Moustafa Sherief Moustafa, Herbert Meier, Heinz Kolshorn, Mohamed Hilmy Elnagdi

**Affiliations:** 1 Department of Chemistry, Faculty of Science; University of Kuwait: Safat; 13060: Kuwait; 2 Institute of Organic Chemistry, Johannes Gutenberg-University, Mainz, Germany

**Keywords:** Aminopyrrole, Pyridazine, Pyrazolopyridazine, Phenacylmalononitrile.

## Abstract

Phenacylmalononitrile **1** reacts with dimethylformamide dimethyl acetal to yield an enaminone which could be readily converted into a pyrrole or an aminopyridazine by treating with ammonium acetate and hydrazine hydrate, respectively. Compound **1** reacted with hydrazine hydrate in ethanol at room temperature to yield the dihydropyridazine **9** as a single product. In refluxing ethanol this product further reacted with hydrazine hydrate to yield the novel dihydropyrazolopyridazinamine **10**.

## Introduction

Malononitrile and malononitrile derivatives are versatile reagents and their chemistry has been studied in the past [1,2,3] and still attracts considerable interest [4,5]. In the past thirty years we have reported several new approaches to a variety of polyfunctional heterocycles utilizing malononitrile or substituted malononitriles as precursors [6,7,8,9,10] and several of these products as been established to act as anti-profiler agents. Very recently we have reported on the utility of benzylmalononitrile as precursor to diaminopyrazoles, diaminoisoxazoles, thiazoles and condensed azoles [11]. In the present paper we report the results of our exploration of the synthetic potential of 2-(2-oxo-2-phenylethyl) malononitrile (**1**) as a heterocycle precursor. This work has allowed us to develop a new route to aminopyrroles and aminopyridazines. The amines formed are of potential utility in the dye industry and as precursors for pharmaceuticals. In addition to that, the reported structures of the reaction products of (2-oxo-2-phenylethyl) malononitrile (**1**) with hydrazine hydrate have been reexamined and corrected in the light of our findings.

## Results and Discussion

**Scheme 1:**
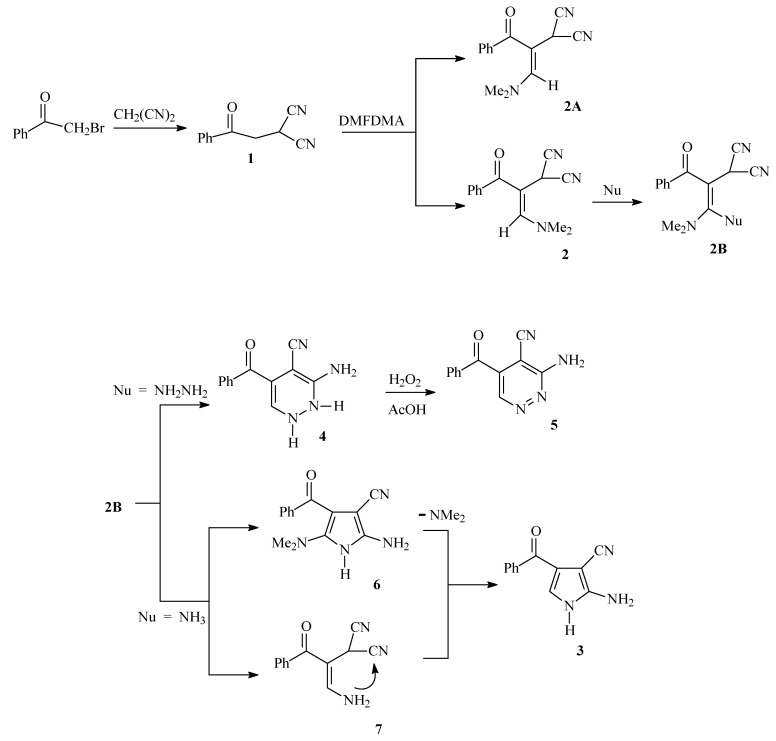
Syntheses of aminopyridazines and aminopyrrole carbonitrile.

Phenacylmalononitrile has been prepared by treating malononitrile with phenacyl bromide. While the literature procedure [12] afforded the desired product in 79 % yield, reaction of phenacyl bromide with malononitrile in ethanolic potassium hydroxide solution gave 2**-**(2-oxo-2-phenylethyl) malononitrile (**1**) in 85% yield. Heating phenacyl bromide and malononitrile in the presence of potassium hydroxide solution in a microwave oven at 85 °C gave an 80% yield of the target product. Reaction of **1** with dimethylformamide dimethyl acetal afforded 2-(3-(dimethylamino)-1-oxo-1-phenylprop-2-en-2-yl) malononitrile (**2**) in 75% yield. Although **2** may also exist in the *Z* form, only **2** (the *E* form) was isolated according to the NMR NOE difference which indicated that the olefinic proton at δ = 8.44 ppm is not sterically proximal to the methylene proton at δ = 7.10 ppm. Refluxing **2** in acetic acid in presence of ammonium acetate afforded the aminopyrrole carbonitrile **3** in 60% yield. On the other hand, reaction of **2** with hydrazine hydrate afforded the aminopyridazine **4** that was readily oxidized to **5** upon treatment with H_2_O_2_ in acetic acid (Scheme 1). It is assumed that initially ammonia adds across the double bond in **2** to yield an intermediate that then looses dimethylamine or alternatively, initially losses dimethylamine and then cyclizes to form **4** (Scheme 1).

**Scheme 2:**
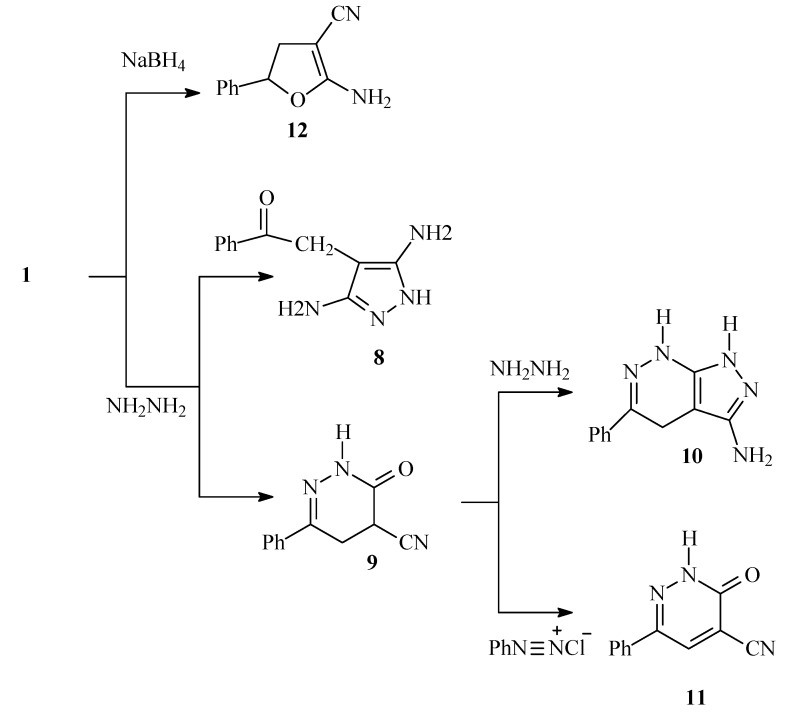
Synthesis of 5-phenyl-4,7-dihydro-1H-pyrazolo[3,4-c]pyridazin-3-ylamine.

It has been previously reported by Abdelrazek *et al* [12] that (2-oxo-2-phenylethyl) malononitrile (**1**) reacts with hydrazine hydrate to yield 4-phenacylpyrazole-3,5-diamine (**8**). Subsequently Elnagdi *et al* [13] have shown that the major product of this reaction was in fact the 3-oxo-6-phenyl-2,3,4,5-tetrahydropyridazine-4-carbonitrile (**9**). Very recently Abdelrazek [14] claimed that in ethanolic solution a pyridazineimine was isolated. These should not be possible as water formed during the reaction would readily hydrolyze readily any imine possibly formed. However, they have also indicated that in refluxing ethanol other product was formed in less than 35% yield and assumed it to be **8**, reported earlier by Abdelrazek *et al.* [12]*.* Now we have found that in ethanol at room temperature **1** reacts with hydrazine hydrate to yield **9** as sole product in 96 % yield. When **9** was refluxed in ethanol with hydrazine hydrate a product of molecular formula C_11_H_11_N_5_ (213.2) was formed. This proved identical with the product obtained by Abdelrazek [12] or the one identified by Elnagdi *et al.* [13]. It thus became clear that **8** had never been isolated and that product believed earlier to be **8** really must have another structure. After inspection of the spectral and analytical data now wish to assign this product as 5-phenyl-4,7-dihydro-1*H*-pyrazolo[3,4-c]pyridazin-3-ylamine (**10**) obtained by further condensation of dihydropyridazine carbonitrile **9** with hydrazine hydrate (Scheme 2). The NMR data of **10** suggest it is in a fast tautomeric equilibrium with the corresponding 2*H* compound.

The dihydropyridazine **9** was readily oxidized to **11** on attempted coupling with benzenediazonium chloride. Compound **11** was also obtained upon oxidizing **9** in an AcOH-H_2_O_2_ mixture (Scheme 2). Reduction of **1** with sodium borohydride in propanol solution afforded 2-amino-5-phenyl-4,5-dihydrofuran-3-carbonitrile (**12**) in 60% yield (Scheme 2). This compound has been synthesized earlier [15] in almost the same way, but no spectral data had been reported. ^1^H-NMR and ^13^C-NMR of now reported for the first time.

We next shifted our interest to an exploration of the chemistry of 2-cyano-5-phenyl-3,5-dioxopentanonitrile, reported by Abdelrazek and Salah El-Din [16] to be formed upon refluxing ethyl benzoyl acetate and malononitrile in ethanolic piperidine solution. Unfortunately, in our hands ethyl benzoylacetate did not react with malononitrile under the reported reaction conditions or even more vigorous ones. Thus it is concluded that 2-cyano-5-phenyl-3,5-dioxopentanonitrile could not have been obtained as reported. It is of interest that in the paper 2-cyano-5-phenyl-3,5-dioxopentanonitrile was claimed to be oil and the authors have not reported analytical data.

## Conclusions

In summary, we have successfully developed a simple route to pyrroles and pyridazines. Moreover, we could show that product claimed earlier to be 4-phenacyl pyrazole-3,5-diamine (**8**) is really 5-phenyl-4,7-dihydro-1*H*-pyrazolo[3,4-c]pyridazin-3-ylamine (**10**).

## Experimental

### General

All melting points are uncorrected and were determined on a Sanyo (Gallaenkamp) instrument. Infrared spectra were recorded in KBr on a Perkin-Elmer 2000 FT–IR system.^1^H-NMR and ^13^C-NMR spectra were determined on a Bruker DPX spectrometer operating at 400 MHz for ^1^H-NMR and 100 MHz for^ 13^C-NMR using in CDCl_3_ or DMSO as solvents and TMS as internal standard; chemical shifts are reported in δ (ppm). Mass spectra were measured on VG Autospec Q MS 30 and MS 9 (AEI) spectrometers, with EI 70 EV. Elemental analyses were measured by means of LEOCHNS-932 Elemental Analyzer. General purpose silica gel on polyester 20 x 20 cm TLC plates with UV indicator were used in TLC experiments. 

### Synthesis of 2-(2-oxo-2-phenylethyl)malononitrile *(**1**)*

A solution of malononitrile (0.66 g, 0.01 mol) and 2-bromo-1-phenylethanone (phenacyl bromide) (1.99 g, 0.01 mol) in ethanol (15 mL) was cooled to 0 ^o^C. Potassium hydroxide (0.84 g, 0.15 mol) was added and the reaction mixture was stirred (followed until completion by TLC using 1:1 ethyl acetate- petroleum ether as eluent). The reaction was carefully quenched with ice-H_2_O and 1M HCl solution, The solid so formed was collected by filtration and recrystallized from ethanol to give a white product; yield 85 %; mp 160-62 °C; Anal. calcd. for C_11_H_8_N_2_O (184.2): C, 71.73; H, 4.38; N, 15.21. Found: C, 71.68; H, 4.35; N, 15.41; IR (KBr): υ_max_ = 2211 (2CN), 1645 (CO); ^1^H-NMR (DMSO): δ, ppm = 4.11 (d, 2H, CH_2_, *J* = 8 Hz), 5.15 (t, 1H, CH, *J* = 8 Hz), 7.56-8.02 (m, 5H, Ar-H); ^13^C-NMR (DMSO): δ, ppm = 194.83, 135.29, 134.69, 129.41 (2C), 128.69 (2C), 114.88 (2CN), 38.75, 18.52. MS: *m/z* (%) 184 (M^+^, 50), 105 (100), 77 (70).

### Synthesis of 2-(3-(dimethylamino)-1-oxo-1-phenylprop-2-en-2-yl)malononitrile *(**2**)*

A mixture of compound **1** (1.84 g, 0.01 mol) and *N*,*N*-dimethylformamide dimethyl acetal (DMFDMA, 1.19 g, 0.01 mol) in toluene (10 mL) was refluxed for 4 h. The reaction mixture was evaporated under reduced pressure yielding a crude product, which was recrystallized from toluene to give a light yellow product; yield 75 %; mp 129-30 °C; Anal. calcd. for C_14_H_13_N_3_O (239.2): C, 70.28; H, 5.48; N, 17.56. Found: C, 70.39; H, 5.50; N, 17.44; IR (KBr): υ_max_ = 2208 (2CN), 1633 (CO); ^1^H-NMR (DMSO): δ, ppm = 3.05 (s, 3H, CH_3_), 3.14 (s, 3H, CH_3_), 7.10 (s, 1H, CH), 7.25-7.42 (m, 5H, Ar-H), 8.44 (s, 1H, CH); ^13^C-NMR (DMSO): δ, ppm = 194.47, 155.33, 134.89, 134.33, 129.43 (2C), 129.04 (2C), 106.93 (2CN), 78.17, 38.31, 37.44, 18.09; MS: *m/z* (%) 239 (M^+^, 100), 210 (15), 197 (45), 140 (25), 134 (90), 119 (20), 107 (15), 77 (15), 57 (25).

### Synthesis of 2-amino-4-benzoyl-1H-pyrrole-3-carbonitrile *(**3**)*

A mixture of compound **2** (2.39 g, 0.01 mol) and ammonium acetate (1 g) in acetic acid (10 mL) was refluxed for 1 h. The mixture cooled and then was poured onto ice-water. The solid thus formed was collected by filtration and recrystallized from petroleum ether to give colorless crystals; yield 60 %; mp 297-98 °C. Anal. calcd. for C_12_H_9_N_3_O (211.2): C, 68.24; H, 4.29; N, 19.89. Found: C, 68.23; H, 4.31; N, 19.79; IR (KBr): υ_max_ = 3350, 3330 (NH_2_), 3134 (NH), 2220 (CN), 1656 (CO); ^1^H-NMR (DMSO): δ, ppm = 7.33-7.80 (m, 8H, Ar-H, NH_2_, D_2_O exchangeable, NH, D_2_O exchangeable), 8.18 (s, 1H, CH); ^13^C-NMR (DMSO): δ, ppm = 189.57, 166.25, 158.65, 154.01, 150.03, 129.23 (2C), 128.76, 124.15 (2C), 102.14, 99.13; MS: *m/z* (%) 211 (M^+^, 100), 184 (80), 155 (80), 141 (25), 114 (10), 105 (25), 77 (45).

### Synthesis of 3-amino-5-benzoyl-1,2-dihydropyridazine-4-carbonitrile *(**4**)*

A mixture of compound **2** (2.39 g, 0.01 mol) and hydrazine hydrate (0.5 g, 0.01 mol) in ethanol (10 mL) was refluxed for 5 h (monitored to completion by TIC using 1:1 ethyl acetate-petroleum ether as eluent). The mixture cooled and then was poured onto ice-water. The solid formed was collected by filtration and recrystallized from ethanol to give a yellow product; yield 65 %; mp 227-29 °C; Anal. calcd. for C_12_H_10_N_4_O (226.2): C, 63.71; H, 4.46; N, 24.76. Found: C, 63.55; H, 4.36; N, 24.89; IR (KBr): υ_max_ = 3409, 3330 (NH_2_), 3080 (NH), 3060 (NH), 2200 (CN), 1608 (CO); ^1^H-NMR (DMSO): δ, ppm = 4.76 (br, 2H, NH_2_, D_2_O exchangeable), 7.39-7.82 (m, 8H, Ar-H), 8.20 (br, 1H, NH, D_2_O exchangeable), 9.09 (br, 1H, NH, D_2_O exchangeable); ^13^C-NMR (DMSO): δ, ppm = 192, 166.59, 160.54, 158.34, 149.83, 129.30 (2C), 129.18, 124.34 (2C), 109.24, 99.67; MS: *m/z* (%) 226 (M^+^, 100), 209 (10), 196 (25), 182 (20), 168 (10), 154 (25), 114 (10), 105 (20), 77 (20).

### Synthesis of compound 3-amino-5-benzoylpyridazine-4-carbonitrile *(**5**)*

A mixture of **4** (2.26 g, 0.01 mol) and hydrogen peroxide 100% (3 mL) in acetic acid (10 mL) was refluxed for 2 h (followed to completion by TLC using 1:1 ethyl acetate-petroleum ether as eluent). The mixture was cooled and then poured onto ice-water. The solid formed was collected by filtration and recrystallized from ethanol to give a dark yellow product; yield 55 %; mp 148-150 °C; Anal. calcd. for C_12_H_18_N_4_O (226.2): C, 64.28; H,3.60; N, 24.99. Found: C, 64.02; H, 3.81; N, 24.75; IR (KBr): υ_max_ = 3380, 3212 (NH_2_), 2281 (CN), 1643 (CO); ^1^H-NMR (DMSO): δ, ppm = 5.56 (br, 2H, NH_2_, D_2_O exchangeable), 7.57 -8.12 (m, 5H, Ar-H), 8.35 (s, 1H, CH); ^13^C-NMR (DMSO): δ, ppm = 191.32, 176.53, 154.54, 143.39, 129.99 (2C), 126.34 (2C), 121.54, 118.46, 111.42, 100.98; MS: *m/z* (%) 224 (M^+^, 75), 206 (15), 196 (50), 180 (25), 164 (10), 154 (35), 128 (10), 105 (45), 77 (50).

### Syntheses of 3-oxo-6-phenyl-2,3,4,5-tetrahydropyridazine-4-carbonitrile *(**9**)* and 5-phenyl-4,7-dihydro-1H-pyrazolo[3,4-c]pyridazin-3-ylamine *(**10**)*.

Procedure 1*:* A mixture of compound **1** (1.84 g, 0.01 mol) and hydrazine hydrate (0.50 g, 0.01 mol) in ethanol (10 mL) was stirred for 8 h at room temperature (followed by TLC until completion using ethyl acetate-petroleum ether 1:1 as eluent). The reaction mixture was poured onto ice-water. The solid product formed was collected by filtration and crystallized from ethanol to give white product **9** in 90% yield.

Procedure 2**:** A mixture of compound **1** (1.84 g, 0.01 mol) and hydrazine hydrate (0.50 g, 0.01 mol) in ethanol (10 mL) was refluxed for 5 h (followed by TLC until completion using ethyl acetate-petroleum ether 1:1 as eluent). The reaction mixture was cooled and poured onto ice-water. The solid product thus formed was collected by filtration and washed with hot ethanol to extract the white product **9**. The residue was crystallized from *N*,*N*-dimethylformamide (DMF) to yield purple crystals of **10**. 

Compound **9**: Yield 35 %; mp 253-55 °C. Anal. calcd. for C_11_H_9_N_3_O (199.2): C, 66.32; H, 4.55; N, 21.09. Found: C, 66.54; H, 4.32; N, 21.30. IR (KBr): υ_max_ = 3234 (NH), 2154 (CN), 1693 (CO); ^1^H-NMR (DMSO): δ, ppm = 3.27 (dd, 1H, *J* = 12.6, 6.0), 3.59 (dd, 1H, *J* = 12.6, 6.0), 4.50 (dd, 1H, *J* = 13.2, 6.0), 7.45-7.79 (m, 5H, Ar-H), 11.49 (br, 1H, NH, D_2_O exchangeable); ^13^C-NMR (DMSO): δ, ppm = 159.81, 148.66, 134.91, 129.91, 126.99 (2C), 125.75 (2C), 116.92 (CN), 29.90, 25.61; MS: *m/z* (%) 199 (M^+^, 100), 170 (15), 155 (15), 140 (15), 115 (25), 103 (80), 77 (35).

Compound **10**: Yield 65 %; mp 288-90 °C. Anal. calcd. for C_11_H_11_N_5_ (213.2): C, 61.96; H, 5.20; N, 32.84. Found: C, 61.77; H, 5.32; N, 32.65. IR (KBr): υ_max_ = 3159, 3012 (NH_2_), 3012 (NH), 2983 (NH); ^1^H-NMR (DMSO): δ, ppm = 3.57 (s, 2H, 4-H), 7.33 (m, 1H, p-H, phenyl), 7.39 (m, 2H, m-H, phenyl), 7.70 (m, o-H, phenyl), 10.11 (br. s, 1H, 7-H, D_2_O exchangeable), the other NH signals are too broad to be localized; ^13^C-NMR (DMSO): δ, ppm = 154.1, 146.7 (C-5), 138.0 (br., C-3a and C-7a), 137.8 (i-C), 128.3 (m-C), 128.1 (p-C), 124.7 (o-C), 77.7 (C-3a), 21.3 (C-4); MS: *m/z* (%) 214 (M^+^, 100), 185 (35), 171 (15), 141 (15), 115 (25), 77 (35).

### Synthesis of 3-Oxo-6-phenyl-2,3-dihydropyridazine-4-carbonitrile *(**11**)*

A cold solution of phenyldiazonium chloride (0.01 mol) was prepared by adding a solution of sodium nitrite (0.7 g into 10 mL H_2_O) to a cold solution of aniline hydrochloride (0.93 g, 0.01 mol of aniline in 5 mL concentrated HC1) with stirring at room temperature. The resulting solution of phenyldiazonium chloride was then added to a cold solution of compound **9 **(1.99 g, 0.01 mol) in ethanol (50 mL) containing sodium acetate (2 g). The reaction mixture was stirred for 1 hr. The solid product formed was collected by filtration and crystallized from ethanol to give a yellow product, yield 70 %; mp 295-97 °C. Anal. calcd. for C_11_H_7_N_3_O (197.2): C, 67.00; H, 3.58; N, 21.31. Found: C, 66.98; H, 3.92; N, 21.65. IR (KBr): υ_max_ = 3217 (NH), 2233 (CN), 1664 (CO); ^1^H-NMR (DMSO): δ, ppm = 7.46-7.91 (m, 5H, Ar-H), 8.85 (s, 1H, CH), 14.04 (br, 1H, NH, D_2_O exchangeable); ^13^C-NMR (DMSO): δ, ppm = 158.78, 148.35, 142.99, 138.34, 135.90, 130.34 (2C), 128.22 (2C), 119.26, 114.66. MS: *m/z* (%) 197 (M^+^, 100), 169 (65), 140 (95), 114 (35), 102 (40), 77 (35), 63 (20).

### Synthesis of 2-amino-5-phenyl-4,5-dihydrofuran-3-carbonitrile *(**12**)*

A solution of compound **1 **(1.84 g, 0.01 mol) in isopropanol (5 mL) was cooled to 0 ^o^C. Sodium borohydride (0.756 g, 0.02 mol) was added and the reaction mixture was stirred for several hours (followed by TLC using ethyl acetate-petroleum ether 1:1 as eluent). If any starting compound was present, more sodium borohydride (up to 0.6 equivalents) was added. The reaction was carefully quenched with ice-H_2_O and 1M HCl solution, extracted with dichloromethane, filtered, and concentrated. The crude product was recrystallized from petroleum ether bp 60-80°C to give colorless crystals; yield 60 %; mp 116-18 °C. Anal. calcd. for C_11_H_10_N_2_O (186.21): C, 70.95; H, 5.41; N, 15.04. Found: C, 71.05; H, 5.36; N, 14.84. IR (KBr): υ_max_ = 3321, 3267 (NH_2_), 2187 (CN); ^1^H-NMR (CDCl_3_): δ, ppm = 2.93 (dd, 1H, *J* = 12.4, 8), 3.313 (dd, 1H, *J* = 12.0, 8.0), 4.84 (br, 2H, NH_2_, D_2_O exchangeable), 5.64 (dd, 1H, *J* = 12.4, 8.0), 7.28-7.44 (m, 5H, Ar-H); ^13^C-NMR (DMSO): δ, ppm = 167.67, 140.60, 128.57 (2C), 128.23, 125.66 (2C), 119.98, 82.28, 46.39, 36.60. MS: *m/z* (%) 186 (M^+^, 100), 169 (45), 143 (80), 115 (95), 106 (15), 77 (45), 63 (15).
